# Effects of Incremental Inspiratory Load on Inspiratory Muscle Activity in Subjects with Chronic Stroke in Comparison to Healthy Controls: A Case–Control Study

**DOI:** 10.3390/jfmk11030269

**Published:** 2026-07-13

**Authors:** Eliete M. Colaço, Alana E. F. da Gama, Lucien P. Gualdi, Jéssica D. M. Fonseca, Daniella C. Brandão, Guilherme A. F. Fregonezi, Armele de Fátima Dornelas de Andrade

**Affiliations:** 1 Department of Physiotherapy, Federal University of Pernambuco, Recife 50740-550, PE, Brazil; eliete.colaco@maisunifacisa.com.br (E.M.C.); alana.elza@ufpe.br (A.E.F.d.G.); daniella.brandao@ufpe.br (D.C.B.); guilherme.fregonezi@ufrn.br (G.A.F.F.); 2 PneumoCardioVascular Lab, Federal University of Rio Grande do Norte, Natal 59078-970, RN, Brazil; lucien.gualdi@ufrn.br (L.P.G.); jessicadanielle00@gmail.com (J.D.M.F.); 3 Faculty of Health Sciences of Trairi, Federal University of Rio Grande do Norte, Santa Cruz 59200-000, RN, Brazil

**Keywords:** stroke, respiratory muscles, threshold limit values, electromyography

## Abstract

**Background**: Inspiratory training after stroke has been used to improve muscle function and strength; however, its acute effects under different inspiratory loads are not well understood. **Aim**: To assess inspiratory muscle activation under different incremental inspiratory loads in individuals with hemiparetic stroke and healthy controls and to compare muscle activation between hemibodies and sexes in post-stroke subjects using surface electromyography. **Methods**: Individuals with stroke and healthy controls were recruited. An incremental inspiratory load test was performed using the Threshold^®^ IMT device, with progressive increases of 15%, 30%, 45%, and 60% of maximal inspiratory pressure (MIP) or up to the maximal device load based. Muscle activation of the scalene, sternocleidomastoid, and lowest intercostal space was recorded bilaterally using surface electromyography. **Results**: The sample consisted of 32 stroke participants (11 females and 21 males) and 14 healthy controls (six females and eight males). Muscle activity was decreased in stroke subjects in comparison to healthy individuals (*p* < 0.05). The paretic side showed decreased muscle activity when compared to the healthy side in all muscles during different incremental loads and maximal inspiratory pressure (*p* < 0.05), mainly in males. Females with stroke showed decreased activity in sternocleidomastoid (15% of MIP) and scalene (15% of MIP and MIP) (*p* < 0.05) compared to controls. Stroke males showed decreased diaphragm activity during 60% or maximal device load of MIP compared to controls (*p* < 0.05). **Conclusions**: Incremental inspiratory loads increased muscle activity in both stroke subjects and controls of both sexes, although in different patterns. Reduced inspiratory muscle activity on the paretic side suggests an influence of stroke on muscular function and should be considered during rehabilitation.

## 1. Introduction

Hemiplegia and hemiparesis are the most common clinical signs in individuals with stroke [1], persisting to chronicity in 40% of the cases [2]. Muscle strength is also impaired in post-stroke subjects, which leads to activity limitation and/or restriction reducing individuals’ functional capacity [3]. Muscle weakness is not limited to upper and lower limbs. Previous studies have shown reduced respiratory muscles strength by one-third to one-half in these subjects in comparison to healthy individuals [4,5]. Alterations of respiratory muscles also lead to impaired breathing pattern and decreased lung volumes [6] and increase the likelihood to chest wall infections [1]. Moreover, changes in respiratory system kinematics and progressive respiratory muscles inefficiency play an important role in the progression of restrictive respiratory disease [6].

Incremental loads are imposed on the respiratory system aiming to increase respiratory muscles strength and endurance [7]. The changes promoted by inspiratory training lead to lung volume [8] and ventilation improvements [9]. Inspiratory muscle training is well known in the treatment and rehabilitation of chronic respiratory and cardiovascular diseases as well as healthy subjects [10]. Although recent evidences support inspiratory training after stroke to improve muscles function and strength [11] its acute effects during different inspiratory loads are not well understood. However, the acute neuromuscular responses to different inspiratory loading levels in individuals remain poorly understood. It is also important to consider potential sex-related differences in respiratory muscle activation. Males and females differ in respiratory muscle mass, thoracic dimensions, chest wall mechanics, and ventilatory strategies, factors that may influence the recruitment of inspiratory muscles during loaded breathing [10].

The assessment of respiratory muscle activity can be performed through surface electromyography (sEMG), a technique that measures muscle activation through its electrical activity. sEMG enables understanding muscle strength generation, motion production, and consequent function, characterizing aspects related to motor control, such as muscle coordination, load and contraction [12].

Beyond reductions in respiratory muscle strength, inspiratory muscle dysfunction after stroke may contribute to clinically relevant complications, including impaired cough efficiency, ineffective secretion clearance, reduced exercise tolerance, postural instability, and an increased risk of respiratory complications. These impairments may negatively affect functional independence and quality of life, highlighting the importance of understanding respiratory muscle behavior and recruitment patterns in this population.

Few studies have shown inspiratory muscle activity after stroke [13,14] considering each hemithorax separately as well as the comparison between sexes. Thus, the aims of the study were to assess inspiratory muscle activation during different incremental inspiratory loads in individuals with hemiparetic stroke and healthy controls and to compare muscle activation of post-stroke subjects between hemibodies and sexes by sEMG. The study hypothesis was that different incremental inspiratory loads would alter inspiratory muscle activation in individuals with stroke and health subjects controls and that these responses would differ according to hemibody and sex.

## 2. Materials and Methods

### 2.1. Subjects

Thirty two individuals of both sexes with stroke diagnosis at least 6 months prior to data collection and 14 non-smoker subjects with preserved lung function were enrolled in the study. The exclusion criteria were: individuals presenting aphasia, facial paralysis, pulmonary diseases, previous stroke history as well as subjects unable to perform the procedures due to blood pressure increase (cut-off point 140/90 mmHg). The study was approved by the Research Ethics Committee of the University of Pernambuco (nº 391/07). All individuals were informed about the study and signed Informed Consent in accordance with the principles of the Helsinki declaration [15].

### 2.2. Study Design

**Figure 1:**
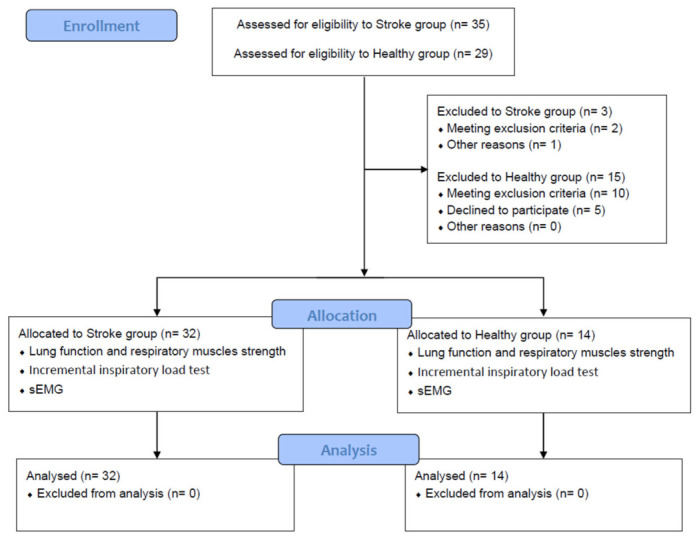
Study flowchart.

This case–control study was performed at the university outpatient clinic. This study was performed in accordance with the “Strengthening the Reporting of Observational Studies in Epidemiology” (STROBE) statement. Initially, all individuals underwent anamnesis and, anthropometric and cardiorespiratory assessment. The following variables were collected: weight, height, arterial pressure (AP), heart rate (HR), respiratory rate (RR) and oxygen saturation (SpO_2_). Lung function, maximal inspiratory pressure (MIP) and maximal expiratory pressure (MEP), minute volume (MV), and tidal volume (VT) were also collected. Incremental inspiratory load test and inspiratory muscles sEMG evaluation were performed simultaneously. Figure 1 illustrates the study flowchart.

### 2.3. Lung Function and Respiratory Muscles Strength

Forced expiratory volume in the 1st second (FEV1) and forced vital capacity (FVC) were measured using a spirometer MicroMedical MicroLoop MK8 (Kent, UK) following the Nacional and Internacional standardization of spirometry technical procedures [16,17]. Predicted values were calculated using previously published reference values for the study population [18].

Respiratory maximal pressures were assessed by measuring inspiratory (MIP) and expiratory (MEP) maximal pressures with a manovacuometer (MV-150 Marshall-Town Instrumentations Industries (El Paso, TX, USA). The subjects were asked to perform MIP from residual volume and MEP from total lung capacity [19]. Reference values were calculated in accordance with previous standardized equations for the studied population [20].

### 2.4. Incremental Inspiratory Load

Incremental inspiratory load test was performed using Threshold^®^ IMT device (Health Scan Products Inc.; Cedar Grove, NJ, USA). The protocol consisted of progressively increasing inspiratory loads corresponding to 15%, 30%, 45%, and 60% of MIP, or until the maximum device load was reached. The loads were applied in a fixed incremental order. Participants breathed against each load for one minute, followed by a three-minute rest between stages. The test was continuously monitored and interrupted if the participant was unable to sustain the inspiratory effort required to overcome the imposed load. Scalene, sternocleidomastoid and lowest intercostal space muscle activity was recorded through sEMG bilaterally during different incremental load tests.

### 2.5. Respiratory Muscles Surface Electromyography

Myoelectric activity was recorded during rest, MIP and MEP maneuvers, and different incremental inspiratory loads (15, 30, 45 and 60% or maximal device load). sEMG was performed following the recommendations of the International Society of Electromyography and Kinesiology [21,22]. Signals were captured using an eight-channel electromyographer (EMG System—Brazil, Sao Paulo, Brazil) with a 20–500 Hz band-pass filter, gain of 1000 and, common-mode rejection ratio greater than 120 dB.

Electromyography evaluation was performed using bipolar technique, with disposable electrodes placed bilaterally over scalene (5 cm from sternoclavicular joint and 2 cm above this point); sternocleidomastoid (5 cm below mastoid process) [9,23], and diaphragm (on the lowest intercostal space, near the costochondral junction) [24]. Supplementary Figure S1 shows the placement of the sEMG electrodes on the evaluated muscles.

sEMG data were captured and stored using the AqDados program (Lynx Tecnologia, São Paulo, Brazil) and analyzed using Matlab^®^, R2009a: Versão 7.8, (Natick, MA USA). To filter cardiac signal, an additional 30 Hz high-pass butterworth filter was used [25]. The signal was analyzed through root mean square (RMS) of captured signals, and standardization was performed from basal respiratory values, i.e., at rest [26]. The activation value of each muscle during quite breathing (without load) has been standardized as equal to 1%, and activation higher or lower than this value was considered as activity increase or decrease, respectively.

### 2.6. Statistical Analysis

The data were described as mean and standard error. Sample normality was analyzed using the Kolmogorov–Smirnov test. Anthropometric characteristics, cardiorespiratory assessment, pulmonary function, and respiratory muscle strength were analyzed using Student’s *t*-test. Intragroup and intergroup analyses were performed using two-way ANOVA and Bonferroni post hoc test. SPSS version 15.0 with a 95% significance level was used for the analysis.

The effect size was calculated considering *p* < 0.05. The mean and standard deviation from electrical activity of scalene, sternocleidomastoid and lowest intercostal space were used. Considering the sample size of 14 subjects in both groups, the results showed statistical power of 0.79 for scalene, 0.9 for sternocleidomastoid and 0.99 for lowest intercostal space (1 − β err. Prob.). Muscles electrical activation effect size was 0.42 for scalene, 0.49 for sternocleidomastoid and 0.81 for lowest intercostal space.

## 3. Results

### 3.1. Anthopometry, Cardiorespiratory, Pulmonary Function, and Respiratory Muscle Strength

Table 1 shows the characterization of the study sample. There was no significant difference regarding age and anthropometric characteristics when we compared subjects with stroke to healthy individuals. Subjects with stroke exhibited higher values in cardiorespiratory variables, except for SpO_2_. Lung function parameters, as well as maximal respiratory pressures, were significantly lower in subjects with stroke compared to controls (*p* < 0.05).

### 3.2. Inspiratory Muscle Activity Comparison Between Paretic and Healthy Sides in Post-Stroke Subjects

**Figure 2:**
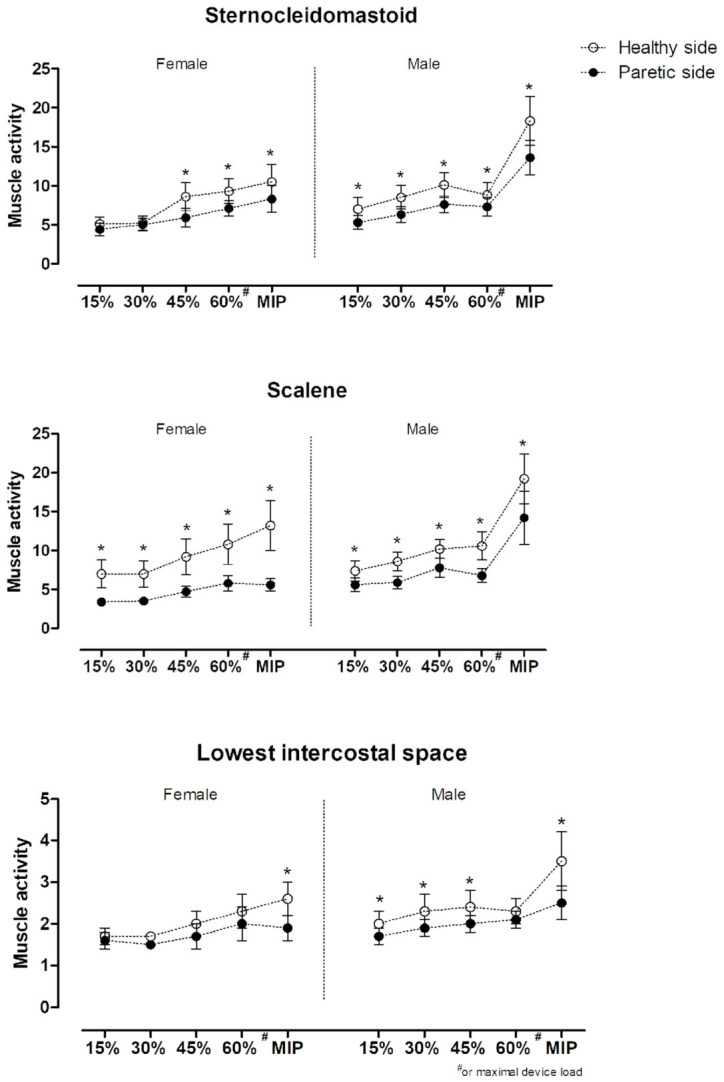
Muscle activity of paretic versus healthy side comparison during different incremental inspiratory loads and maximal inspiratory load (MIP) in post-stroke individuals according to sex. Open symbol: healthy side; closed symbol: paretic side. Intra and intergroup analysis performed by two-way ANOVA; * *p* < 0.05.

Post-stroke individuals showed decreased activity of sternocleidomastoid (45 and 60% maximal device load [*p* < 0.001, CI 1.16–4.24 and *p* < 0.01, CI 0.66–3.73] versus 15, 30, 45 and 60% [*p* < 0.001, CI 0.34–3.06, *p* < 0.001, CI 1.14–3.86 and *p* < 0.05, CI 3.34–6.06] maximal device load of MIP for females and males, respectively) in the paretic side in comparison to the healthy side. Scalene muscle activity was decreased in the paretic side during all incremental loads (15, 30, 45, and 60%/maximal device load of MIP) in both sexes [*p* < 0.001, CI 1.63–5.56, *p* < 0.001, CI 1.53–5.46, *p* < 0.001, CI 2.53–6.46 and *p* < 0.001, CI 3.03–6.96, respectively for females and *p* < 0.001, CI 0.34–3.27, *p* < 0.001, CI 1.24–4.17, *p* < 0.001, CI 0.94–3.87 and *p* < 0.001, CI 2.34–5.27, respectively for males]. The lowest intercostal space activity was decreased during 15, 30 and 45% incremental loads in the paretic side of the male sexes [*p* < 0.05, CI 0.01–0.59, *p* < 0.01, CI 0.11–0.69 and *p* < 0.01, CI 0.11–0.69] while there was no significant difference in the female gender. When we compared paretic side to healthy side during MIP we found decreased activity of all muscles in the paretic side in both sexes [sternocleidomastoid *p* < 0.05, CI 3.34–6.06, scalene *p* < 0.001, CI 5.54–6.47, and the lowest intercostal space *p* < 0.001, CI 0.71–1.29, for females and sternocleidomastoid *p* < 0.01, CI 0.66–3.74, scalene *p* < 0.001, CI 5.63–9.56 and lowest intercostal space *p* < 0.001, CI 0.37–1.03, for males] (Figure 2).

### 3.3. Inspiratory Muscle Activity Comparison Between Subjects with Stroke and Healthy Controls

**Figure 3:**
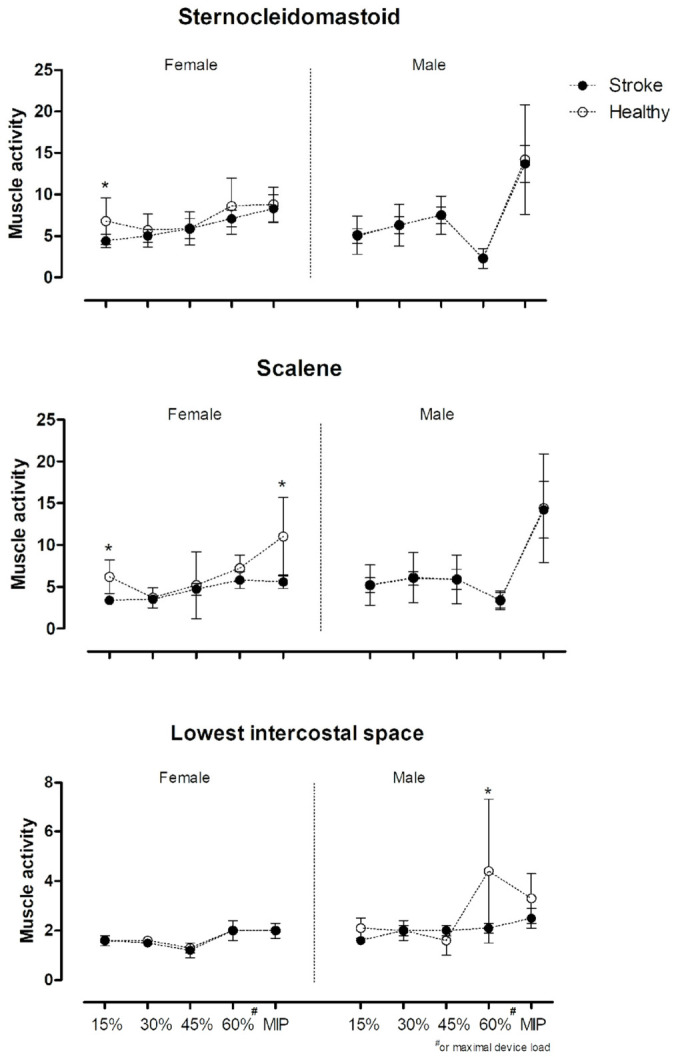
Muscle activity comparison between post-stroke subjects and healthy individuals during different incremental inspiratory loads and maximal inspiratory load (MIP) according to sex. Open symbol: healthy individuals; closed symbols: post-stroke subjects. Intra and intergroup analysis performed using two-way ANOVA; * *p* < 0.05.

Comparing female subjects with chronic stroke to healthy individuals, we found significant decreased activity in sternocleidomastoid and scalene muscles during 15% of MIP (*p* < 0.05, IC 0.08–4.72 and *p* < 0.05, IC 0.33–5.27, respectively) in the stroke group. Scalene also showed significant difference during MIP (*p* < 0.001, IC 2.93–7.87). There was no significant difference in lowest intercostal space activity between stroke females and controls. Stroke males only showed significant decrease in lowest intercostal space activity during 60%/maximal device load of MIP in comparison to healthy controls (*p* < 0.05, IC 1.48–3.12) (Figure 3).

### 3.4. Inspiratory Muscle Activity Comparison Between Sexes

**Figure 4:**
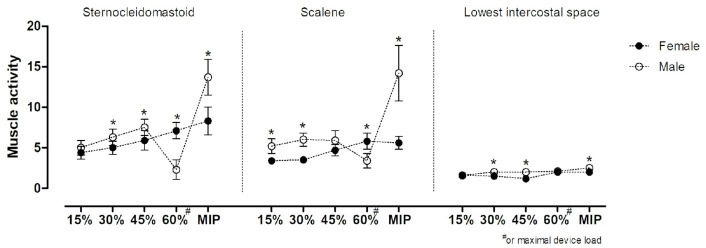
Muscle activity comparison between paretic side in post-stroke males and females paretic side during incremental inspiratory loads and maximal inspiratory load (MIP). Open symbol: males; closed symbol: females. Intra and intergroup analysis performed using two-way ANOVA; * *p* < 0.054.

When we compared the paretic side according to sex we found that males showed increased activity for stermocleidomastoid, scalene and lowest intercostal space muscles during different incremental loads of MIP and MIP in comparison to females [sternocleidomastoid in 30% *p* < 0.05, CI 0.04–2.56, 45% *p* < 0.01, CI 0.44–2.96 and MIP *p* < 0.001, IC 4.04–6.56; scalene in 15% *p* < 0.001, CI 0.76–3.64, 30% *p* < 0.01, CI 0.96–3.84 and MIP *p* < 0.001, IC 7.16–10.04; and the lowest intercostal space in 30% *p* < 0.001, CI 0.15–0.65, 45% *p* < 0.05, CI 0.05–0.55 and MIP *p* < 0.001, IC 0.35–0.85] and a reduction in the activity of the sternocleidomastoid and scalene muscles at 60% load [sternocleidomastoid *p* < 0.001, CI −6.05:−3.55 and scalene *p* < 0.01, CI −3.84:−0.96 respectively] (Figure 4).

## 4. Discussion

This study assesses muscle activation during different incremental inspiratory loads in individuals with hemiparetic stroke and healthy controls and comparing myoelectrical activity of post-stroke subjects between hemibodies and sexes through sEMG. Stroke subjects showed lower maximal respiratory pressures and lung function in comparison to healthy controls. Moreover, reduction in inspiratory muscle electrical activity was found at the paretic side of post-stroke subjects; however different inspiratory incremental loads were able to increase muscles activity in both sides, differentially. Differences in muscle activation were also found when we compared subjects according to sex. However, the mechanisms underlying these sex-related differences cannot be determined from the present data, since thoracoabdominal motion, respiratory kinematics, and muscle morphology were not directly assessed.

The electrical activity recorded from the diaphragmatic region exhibited considerable variability and inconsistent activation patterns, which limited the interpretation of these findings. Although the electrode placement used in the present study has been previously described for the non-invasive assessment of diaphragmatic activity, signal contamination from adjacent inspiratory muscles, particularly the intercostal muscles, cannot be completely excluded. Therefore, the recorded signal should be interpreted as reflecting activity from the diaphragmatic region rather than isolated diaphragmatic activation. This methodological limitation may have contributed to the absence of significant variations in muscle activity observed in our results.

In a study performed by Fugl-Meyer et al. [27] authors found reductions of 40% and 60% of MIP and MEP in subjects with hemiplegia, respectively. Another study performed by Teixeira-Salmela et al. [14] also found a reduction of 21% in MIP and 10% in MEP in post-stroke population, which corroborates with our results as we found decreased values for MIP and MEP in males (25% and 46%) and females (30.8% and 58.5%), respectively. Stroke subjects also showed lower pulmonary volumes compared to healthy individuals in both sexes in our study which corroborates with the study of Tomczak et al. [28] that showed decreased pulmonary volumes in hemiparetic individuals of both sexes. Previous studies showed that post-stroke individuals tend to present low functional capacity—40% lower than sedentary individuals of the same sex and age [14,29]. Other studies have reinforced the theory that decreased volumes and flows in these individuals are due to muscle weakness, which may cause inability to generate or maintain normal respiratory pressures [30].

In a study performed by de Andrade et al. [23] authors have found different increases in muscle activity during inspiratory muscle training using threshold devices in subjects with chronic obstructive pulmonary disease (COPD) and healthy elderly after a six-day training period. Although our population is different from the previous authors’ and we have assessed subjects on a single session we were not able to find great differences in inspiratory muscle activation between subjects with stroke and healthy controls with exception to 15% of MIP in sternocleidomastoid and scalene muscles in which the myoelectrical activity was significantly greater in healthy females in comparison to females who underwent stroke. Such differences may be explained due to the different populations and methodological characteristics of the studies.

Besides the increase in myoelectrical activity in both groups when we compared hemiparetic side to healthy side we found significant decrease in inspiratory muscle activity in the hemiparetic side in comparison to the healthy side. The greater activity in the healthy side may be explained by the fact that although the imposed load which increments muscle activity the asymmetry is maintained as well as the increased muscle tone and spasticity on the lesion side [31].

Several studies have shown an impairment of lowest intercostal space after stroke [27,32,33]. Decreased lowest intercostal space activity and paresis were also observed in the hemiparetic side [30,32], and paresis [34,35]. In this study a significant difference between hemiparetic and healthy sides during incremental loads was found only in the male sex. The lesion side may explain such difference observed among study results. De Almeida et al. [36] have shown greater respiratory impairment in right hemiplegia compared to left hemiplegia.

We have also observed that males showed greater muscle activation in comparison to females in the paretic side during different inspiratory incremental load, which should be taken into consideration during the pulmonary rehabilitation. A possible explanation for such difference relates to the anatomical and physiological differences between sexes. Published data suggest a stronger contribution of the upper thoracic cavity muscles during inspiration in females compared to males due to lowest intercostal space positioning in women [36]. Moreover, upper thoracic cavity muscles tend to have biomechanical advantages when compared to the lowest intercostal space, which due to shorter length and higher position may work in disadvantage during maximal or resisted inspiration [37].

Limitations of this study should be acknowledged. Stroke severity was not assessed, making it impossible to determine whether the degree of neurological impairment influenced inspiratory muscle activation patterns. Additionally, subgroup analyses according to sex and hemibody should be interpreted with caution, as the sample size was not specifically calculated for these comparisons and may have limited statistical power. Furthermore, several potential confounding factors, including physical activity level, body mass index, medication use, trunk impairment, cognitive status, respiratory pattern, and stroke lesion characteristics, were not controlled. Consequently, the observed differences between groups should be interpreted with caution.

Although surface electromyography is a widely used non-invasive technique, the possibility of crosstalk from adjacent muscles cannot be completely excluded. In addition, EMG normalization was performed relative to resting breathing activity, which may not fully account for interindividual differences in muscle recruitment strategies. Breathing patterns were not strictly controlled during the inspiratory loading protocol, and variations in respiratory timing and depth may have influenced muscle activation responses. Moreover, although rest intervals were provided between loading stages, the potential influence of fatigue during the incremental loading protocol cannot be entirely ruled out. Consequently, the observed differences between groups should be interpreted with caution. Additionally, respiratory muscle activation assessed through sEMG may have been influenced by differences in breathing strategy, including breathing frequency, inspiratory flow, tidal volume, and thoracoabdominal movement. Since these variables were not controlled, it is not possible to determine the extent to which altered respiratory mechanics contributed to the observed differences in muscle activation patterns between groups.

Although the study shows some limitations such as heterogeneity among the assessed subjects in terms of stroke chronicity, affected side and sex, we believe that the results have shown important clinical implications as we grouped these individuals separately for the analysis. The Threshold^®^ IMT device also shows some limitations as it limits imposed loads due to its maximal pressure of 41 cmH_2_O. Thus, 60% of MIP comparisons were made considering the percentage load or maximal device load mainly in the healthy controls.

## 5. Conclusions

Inspiratory muscles’ electric activity reduction was detected in subjects with chronic stroke. In general, the use of different incremental loads was able to significantly increase muscles’ activity in subjects with stroke and healthy individuals of both sexes, differently. Moreover, reduced activity of inspiratory muscles in paretic side suggests the influence of the disease on muscular activity and should be considered during rehabilitation.

## Figures and Tables

**Table 1:** Anthropometric, lung function and maximal respiratory pressure comparison between post-stroke subjects and healthy controls.

	Female				Male			
	Stroke (*n* = 11)		Healthy (*n* = 6)		Stroke (*n* = 21)		Healthy (*n* = 8)	
	Mean	Error	Mean	Error	Mean	Error	Mean	Error
Age (years)	52.4	3.9	49.5	5.4	59.8	2.7	53.8	3.2
Weight (Kg)	58.8	3.1	64.9	5.7	71.3	3.4	81.8	6.9
Height (m)	1.6	0	1.6	3	1.7	0	1.7	0
BMI (Kg/cm^2^)	23.6	1.2	26.8	1.3	25.2	1	28.6	1.8
SBP (mmHg)	124.5 *	3.7	119.2	5.2	127.9 *	3.5	131.9	6
DBP (mmHg)	85 *	2	79.2	3.3	84 *	2.7	88.1	5.3
HR (bpm)	86 *	3.8	70.5	2.1	78.9 *	2.6	80.8	5.9
SPO_2_ (%)	97	0.7	98.3	0.4	97.7	0.2	97.1	0.5
RR (bmp)	18.7 *	1.6	15.5	2.2	16.7 *	1.4	12.4	1.7
MIP (cmH_2_O)	54.9 *	4.5	84	7.5	75 *	5.2	101.5	8.5
MEP (cmH_2_O)	34.5 *	3.6	69.7	3.8	60.1 *	3.7	95.3	5.4
V_T_ (l)	0.5 *	0	0.6	0.1	0.6 *	0.1	0.7	0.1
VC (l)	1.9 *	0.2	2.7	0.4	2.8 *	0.2	3.7	0.2
FEV_1_%	69.5 *	5.9	93.3	4.2	73.8 *	3.8	86	3.9
FVC%	67.3 *	5.5	94.8	7.8	69.6 *	3.7	89.7	5.4
FEV_1_/FVC%	103.5 *	2.7	100	5.4	106.4 *	2.3	96.6	5.8

BMI: body mass index; SBP: systolic blood pressure; DBP: diastolic blood pressure; HR: heart rate; SPO_2_: saturation of peripheral oxygen; MIP: maximal inspiratory pressure; MEP: maximal expiratory pressure; RR: respiratory rate; Vt: tidal volume; VC: vital capacity; FEV_1_: forced expiratory volume on first second; FVC: forced vital capacity; * *p* < 0.05 (Student’s *t*-test: comparing stroke and healthy subjects).

## Data Availability

The data can be available upon request to the corresponding author.

## Supplementary Materials

The following supporting information can be downloaded at: https://www.mdpi.com/article/10.3390/jfmk11030269/s1.
