# Supplementary material for: Effects of Incremental Inspiratory Load on Inspiratory Muscle Activity in Subjects with Chronic Stroke in Comparison to Healthy Controls: A Case–Control Study
Source: J Funct Morphol Kinesiol. 2026 Jul 13;11(3):269. doi: 10.3390/jfmk11030269 (PMC13398174; doi:10.3390/jfmk11030269)
Supplement: Supplementary file 1 [file jfmk-11-00269-s001.zip › jfmk-4329984-supplementary.pdf]

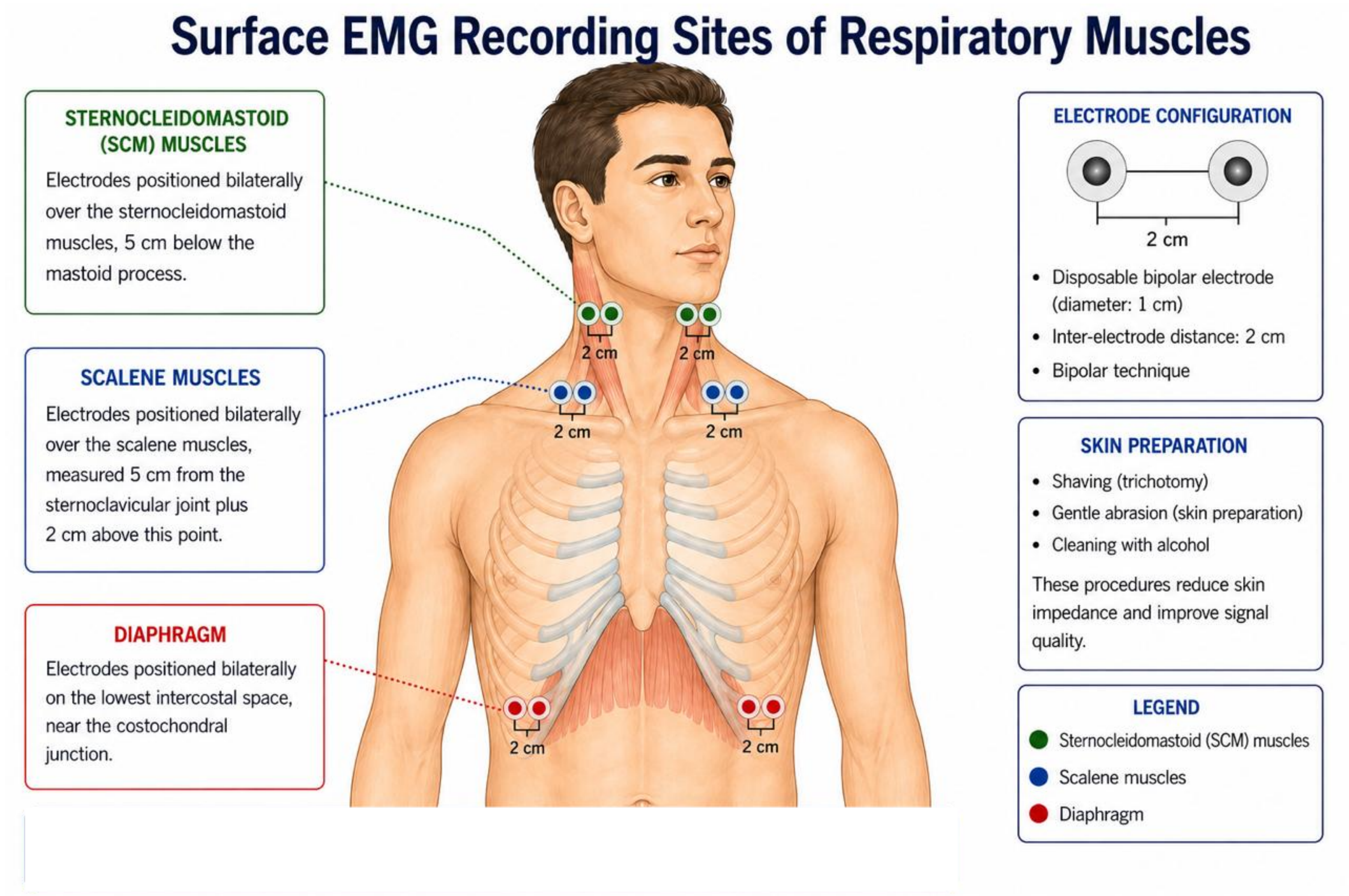

Figure S1. Surface electromyography (sEMG) recording sites of the respiratory muscles. Bipolar disposable surface electrodes (1 cm diameter, 2 cm inter-electrode distance) were positioned bilaterally over the sternocleidomastoid (SCM), scalene, and diaphragm muscles. SCM electrodes were placed 5 cm below the mastoid process. Scalene electrodes were positioned 5 cm from the sternoclavicular joint and 2 cm superior to this point. Diaphragm electrodes were placed bilaterally over the lowest intercostal space near the costochondral junction. Before electrode placement, the skin was prepared by shaving (when necessary), gentle abrasion, and cleaning with alcohol to reduce skin impedance and improve signal quality. All recordings were performed using a bipolar electrode configuration.
